# MPIG6B Gene-Related Myelofibrosis: A Rare Inherited Disease That Is Frequently Described in Arab Population

**DOI:** 10.1055/s-0044-1779697

**Published:** 2024-02-23

**Authors:** Leen Jihad Attar, Almothana Alelaimat, Alaa Alshorman, Tariq N. Aladily

**Affiliations:** 1 Department of Hematopathology, The University of Jordan, Amman, Jordan; 2 Department of Hematology, Ministry of Health, Amman, Jordan

**Keywords:** MPIG6B, myelofibrosis, thrombocytopenia, anemia, hepatosplenomegaly

## Abstract

The megakaryocyte and platelet inhibitory receptor gene G6P (MPIG6B) is located on chromosome 6p21.33. It encodes G6b-B; an inhibitory receptor expressed on the surface of platelets. It regulates platelets production, aggregation, and activation. We describe a case of a 31-year-old man who presented with a long history of thrombocytopenia, anemia, and hepatosplenomegaly. The patient received multiple blood transfusions and his clinical course was stable. A bone marrow biopsy showed morphologic features similar to primary myelofibrosis. Whole exome sequencing study was performed and revealed homozygous pathogenic mutation in exon 2 of MPIG6B gene (c.324C > A, p.Cys108Ter) that is the second reported case in literature. In this report, we describe the main clinical and pathologic features of this disease and review the literature of previously documented cases.

## Introduction

The megakaryocyte and platelet inhibitory receptor gene G6P gene (MPIG6B) is located on chromosome 6p21.33; it encodes an inhibitory receptor expressed on the surface of platelets; G6b-B. It regulates platelets production, aggregation, and activation. Loss of function of G6b-B results in thrombocytopenia, anemia, and myelofibrosis early in life, 1 and that has been replicated in female and male MPIG6B mutated mice that show low platelet count, larger platelet volume, and a higher number of bone marrow megakaryocytes, as the absence of G6b-B led to a reduction in surface expression levels of platelet membrane glycoproteins. 2 3 The first such case was described in 2016 in which four siblings manifested at early childhood with persisting thrombocytopenia and anemia. 1

## Case Report

**Fig. 1:**
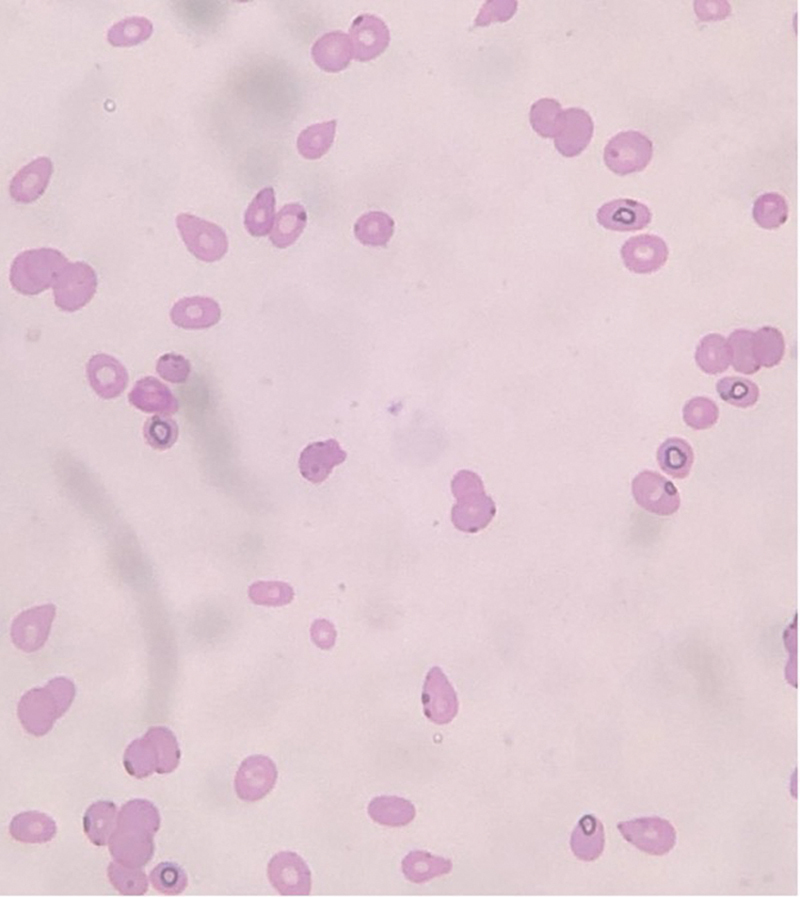
Peripheral blood smear: Tear-drop red blood cells and very few large-size platelets (Wright-Giemsa stain, 1000× magnification).

**Fig. 2:**
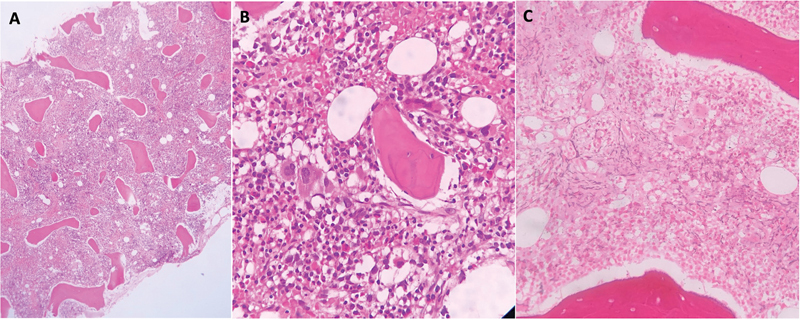
( **A** ) Bone marrow biopsy: Low-power view shows hypercellularity and sclerosis of bone trabeculae (hematoxylin and eosin stain. × 200). ( **B** ) Bone marrow biopsy: High-power view shows megakaryocytic hyperplasia with abnormal morphology along with osteosclerosis (hematoxylin and eosin stain. × 500). ( **C** ) Bone marrow biopsy: Fine reticulin fibers (reticulin stain).

A 31-year-old man presented to our hospital with fever and generalized weakness. Upon physical examination, hepatosplenomegaly was found along with short stature for his age and abnormal skeleton. Complete blood count revealed anemia (hemoglobin concentration of 8 g/dL), mean cell volume of 89.5 fL, white blood cell counts of 6.09 × 10 ^9^ /L with a normal differential, platelets count of 54,000 /μL, and mean platelet volume of 18.5 fL. Peripheral blood smear showed tear-drop red blood cells and very few large-size platelets ( Fig. 1 ). Bone marrow core biopsy revealed hypercellularity and megakaryocytic hyperplasia with abnormal morphology along with osteosclerosis ( Fig. 2 ). Reticulin special stains showed fine fibrosis.

Whole exome gene sequencing study was performed and revealed a pathogenic homozygous mutation in MPIG6B gene c.324C > A (p.Cys108Ter) located on exon 2. The patient is an offspring of a third-degree consanguineous marriage with no family history of a similar illness. The diagnosis of MPIG6B-related myelofibrosis was established, and the patient is planned to receive hematopoietic stem cell transplant as nowadays, bone marrow transplant is the only curative treatment for myelofibrosis. 4

A verbal consent to report the case was given from the patient.

## Discussion

The p.Cys108Ter mutation is predicted to cause loss of normal protein function through protein truncation. This variant is a stop gained variant that occurs in an exon of MPIG6B upstream of where nonsense-mediated decay is predicted to occur and was previously classified as pathogenic, indicating that the region is critical to protein function. Homozygous mutation in the MPIG6B gene on chromosome 6p21 causes MPIG6B-related myelofibrosis that is a rare disease in which patients usually manifest with thrombocytopenia, anemia, and bone marrow fibrosis early in life. The disease was previously described in seven articles with a total of 20 patients. Demographically, male to female ratio was 1.2:1. Most patients were Arabs (frequency of 80%), 2 were Asians, 1 was white, and 1 was South Asian. All the cases were alive except for a single patient who died due to complications of hematopoietic stem cell transplant. This data is illustrated in Table 1 .

**Table 1:** Previous literature on MPIG6B-related myelofibrosis

Reference	Date	Gender	Age of symptoms	Race	Presentation	HMZ MPIG6B mutation	Management	Outcome
Melhem et al 1 #4 cases	2016	3 M 1 F	Early childhood	Arab	T & A	c.324C > A (p.Cys108Ter)	Occasional platelet Transfusion No response to steroids	Alive
Hofmann et al 5 #9 cases	2018	6 M 3 F	Early childhood	Arab	MF	1) c.61_61 + dup 2) c.149dup (p.Ala52GlyfsX128) 3) c.469G > A (p.Gly157Arg)	Frequent platelet transfusion Occasional RBC transfusion HSCT ( *n* = 3)	*n* = 1 died from HSCT complication, the others are alive
Chen et al 6	2019	M	10-month-old	Chinese	Pallor, splenomegaly and resistant hemocytopenia	c.392delC,p.P134Lfs*10	Blood and platelet transfusions and antiinfective therapy.	Alive
Saliba et al 7	2020	F	26	European	Evans syndrome at five, symptoms of easy bruising, myelofibrosis	c.469G > A (p.Gly157Arg)	Systemic glucocorticoids, IV immunoglobulin therapy and cyclophosphamide & vincristine, splenectomy	Alive
Batis et al 8 #2 cases	2021	2 F	Less than 1 year	Arab	T & A Epistaxis	c.523C > T (p.Arg175Ter) c.149dup (p.Ala52GlyfsX128)	Platelet transfusion, occasional RBC transfusion, splenectomy ( *n* = 1), steroid	Alive
Khan et al 9	2022	M	21	Indian	T & A Splenomegaly	c.132G > A (p.Trp44Ter)	Steroids, platelet transfusion, romiplostim	Alive
Wang et al 10	2022	F	14	Chinese	Cutaneous petechia, pallor, T & A	c.420T > A(p.Tyr140Ter)	HSCT	Alive
Our case	2023	M	Early childhood	Arab	T & A MF Fever & generalized weakness	c.324C > A (p.Cys108Ter)	Steroids, recurrent blood transfusion	Alive

Abbreviations: A, anemia; F, female; HSCT, hematopoietic stem cell transplant; IV, intravenous; M, male; MF, myelofibrosis; MPIG6B, megakaryocyte and platelet inhibitory receptor gene G6P; RBC, red blood cell; T, thrombocytopenia.

Primary myelofibrosis is the main differential diagnosis for MPIG6B-related myelofibrosis as the diseases causes anemia, splenomegaly, and bone marrow fibrosis. However, patients are usually old, commonly have thrombocytosis and JAK2 mutation, and the disease is progressive with a poor outcome. Thus, distinction between primary myelofibrosis and MPIG6b-related myelofibrosis is essential.

## Conclusion

MPIG6B-related myelofibrosis is a rare disease that was described in Arabs. The course of the disease is milder than primary myelofibrosis. Patients present with thrombocytopenia in contrast to patients with primary myelofibrosis present with thrombocytosis. Genetic studies are essential to establish the diagnosis.
